# Supplementary figures and images for: Distinct neuronal types contribute to hybrid temporal encoding strategies in primate auditory cortex
Source: PLoS Biol. 2022 May 25;20(5):e3001642. doi: 10.1371/journal.pbio.3001642 (PMC9132345; doi:10.1371/journal.pbio.3001642)

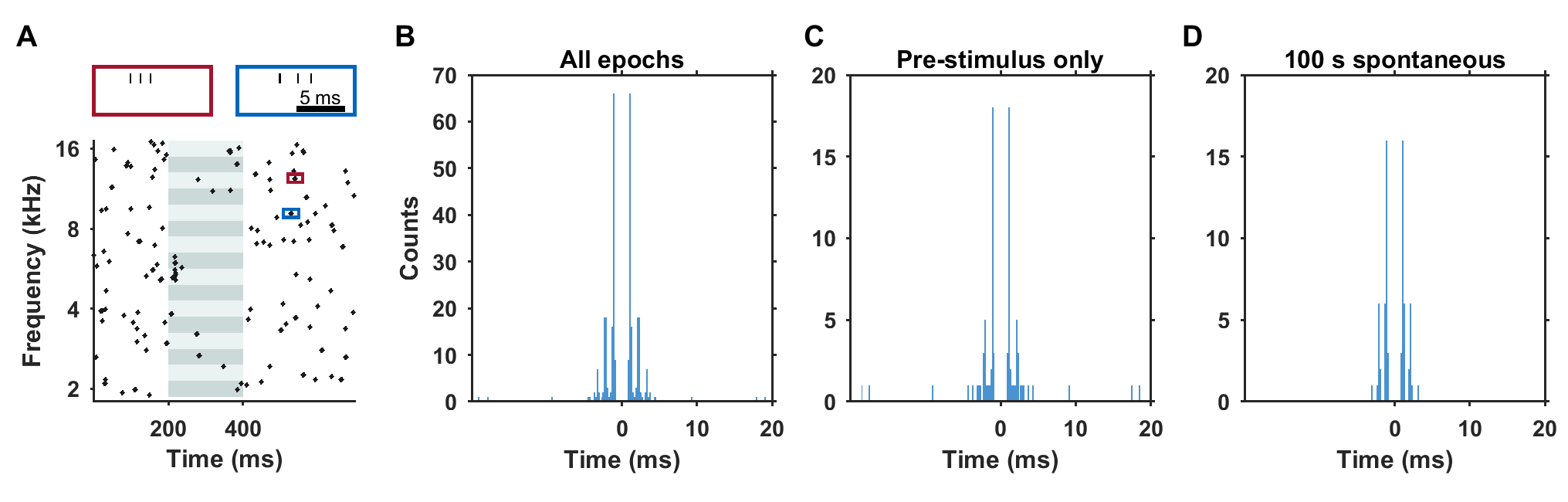

Supplement: S1 Fig — (A) Raster plot of an example bursting unit (M117B0636ch4) in response to tones. A short-latency transient response is seen at 5.3 kHz. Rapid and brief bursts occurred throughout the prestimulus, stimulus, and poststimulus periods, with 2 expanded bursts shown in the red and blue boxes. (B) Autocorrelogram (0.2 ms resolution) calculated from the entire response shows bursting behavior with a peak at 1.1 ms. (C) Autocorrelogram calculated from only the prestimulus periods is also maximal at 1.1 ms. (D) A 100 second-long segment of spontaneous activity was also recorded for this unit and the same spike timing properties can be observed, with a peak at 1.1 ms. Data underlying this figure can be found in S2 Data. (TIF) [file pbio.3001642.s001.tif]

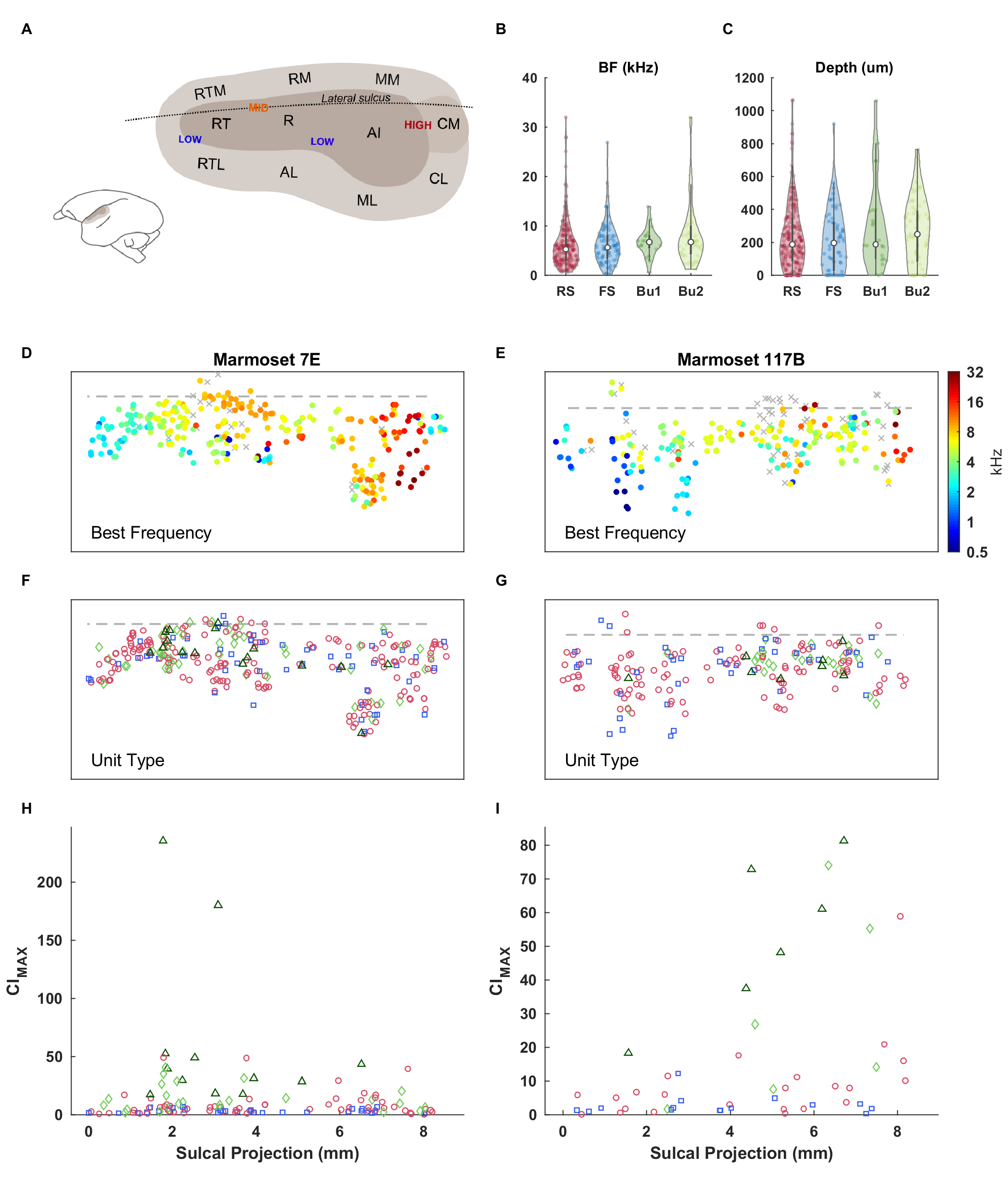

Supplement: S2 Fig — (A) Schematic showing location of auditory cortex along the lateral sulcus of the left hemisphere of the marmoset brain and cortical areas within the core region (dark shading) and belt region (light shading), based on (1–3). Within the core, the tonotopy experiences a low frequency reversal at the lateral border between AI and R, and a mid-frequency reversal at the medial border between R and RT. Recordings were made along the length of the lateral sulcus, primarily in core areas AI, R, and RT, with some likely inclusion of anterior and caudal belt. (B and C) BF and recording depth distributions were generally overlapping for the various unit types (RS, red circles; FS, blue squares; Bu1, dark green triangles; and Bu2 light green diamonds), and should not be a confounding cause for consistent unit type differences observed between unit types. Depths are expressed relative to the first spiking unit encountered from a superficial approach and were biased toward superficial layers due to the long recording times spent with each unit. One-way ANOVAs did not show a statistically significant difference in BF or depth between at least 2 groups (F(3,329) = 1.97, p = 0.12 and F(3,355) = 1.6, p = 0.19). (D andE) Maps of best frequencies of recorded units in the 2 marmosets used in this study, spanning from the low frequency region of anterior RT to the high frequency region of posterior AI. See (H and I) for scale. A small jitter was added to offset multiple units within the same track for visibility. Light gray x’s indicate units that could not be well driven by sound. (F and G) Unit types were distributed throughout recorded areas. For instance, Bu1 units (dark green triangles) were interleaved with other unit types. (H and I) When units were projected onto the sulcal axis, bursting units, and in particular Bu1 units, had higher CImax values regardless of anterior-posterior location. Data underlying this figure can be found in S2 Data. AI, primary auditory cortex; AL, ante [file pbio.3001642.s002.tif]

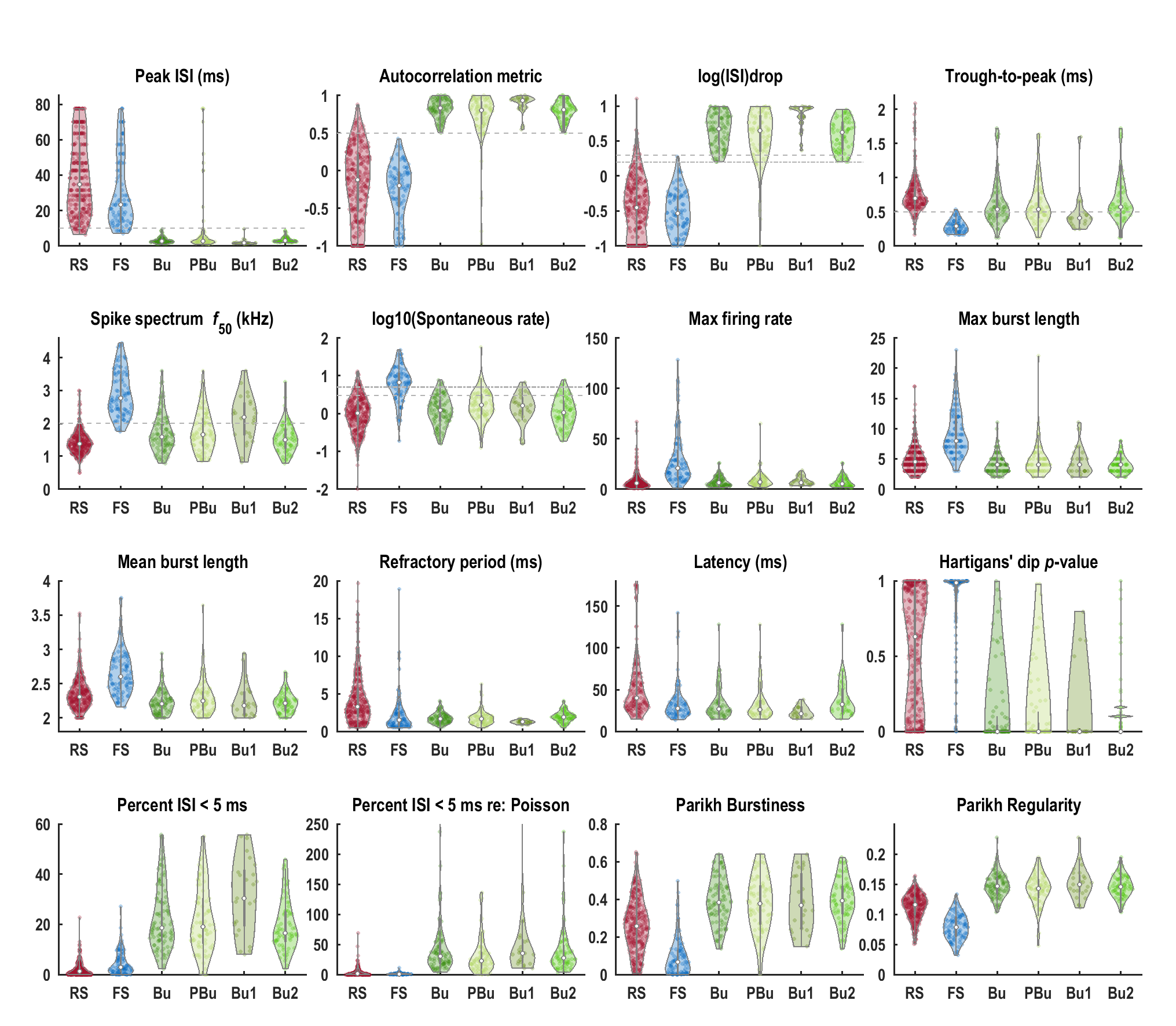

Supplement: S3 Fig — The first 6 properties were used for classification by criteria and the criteria boundaries are shown in gray lines (see Methods). The other properties were not used in making the classification and include basic properties and additional properties we explored for identifying bursting (see Methods). Unit type was determined by criteria, or solely based on prestimulus logISIdrop for PBu. Bu1 and Bu2 are also shown separately. The consensus criteria meant that the cutoff for a single property was often “soft,” as evidenced by the tails of some distributions crossing over the dividing lines. FS units had (1) spikes with shorter tTTP and larger f50 values; (2) higher spontaneous and maximum driven rates to tones; (3) clearly unimodal ISI histograms according to Hartigans’ dip test; and (4) a propensity for firing strings of spikes (high max “burst” length, with burst defined as consecutive ISI values between 0.5 and 1.5 times the mode of the ISI). Bursting units were characterized by (1) very short peak ISI values reflecting the bursting interval; (2) differences in the autocorrelogram metric, logISIdrop, and the percent of ISI less than 5 ms; (3) indication of bimodality on Hartigans’ dip test; and (4) bursts with a smaller max and mean burst length (fewer spikes per burst). RS units had (1) long tTTP and lower f50 values; (2) relatively long calculated refractory periods; and (3) longer minimum response latencies. Compared with Bu2 units, Bu1 units had higher values of the autocorrelation metric and logISIdrop, narrower spikes, shorter refractory periods, and shorter latencies. Three outliers with very large refractory periods are cropped out. Maximum firing rate was the maximum mean rate during a stimulus response window. Data underlying this figure can be found in S2 Data. FS, fast-spiking; ISI, interspike interval; RS, regular-spiking. (TIF) [file pbio.3001642.s003.tif]

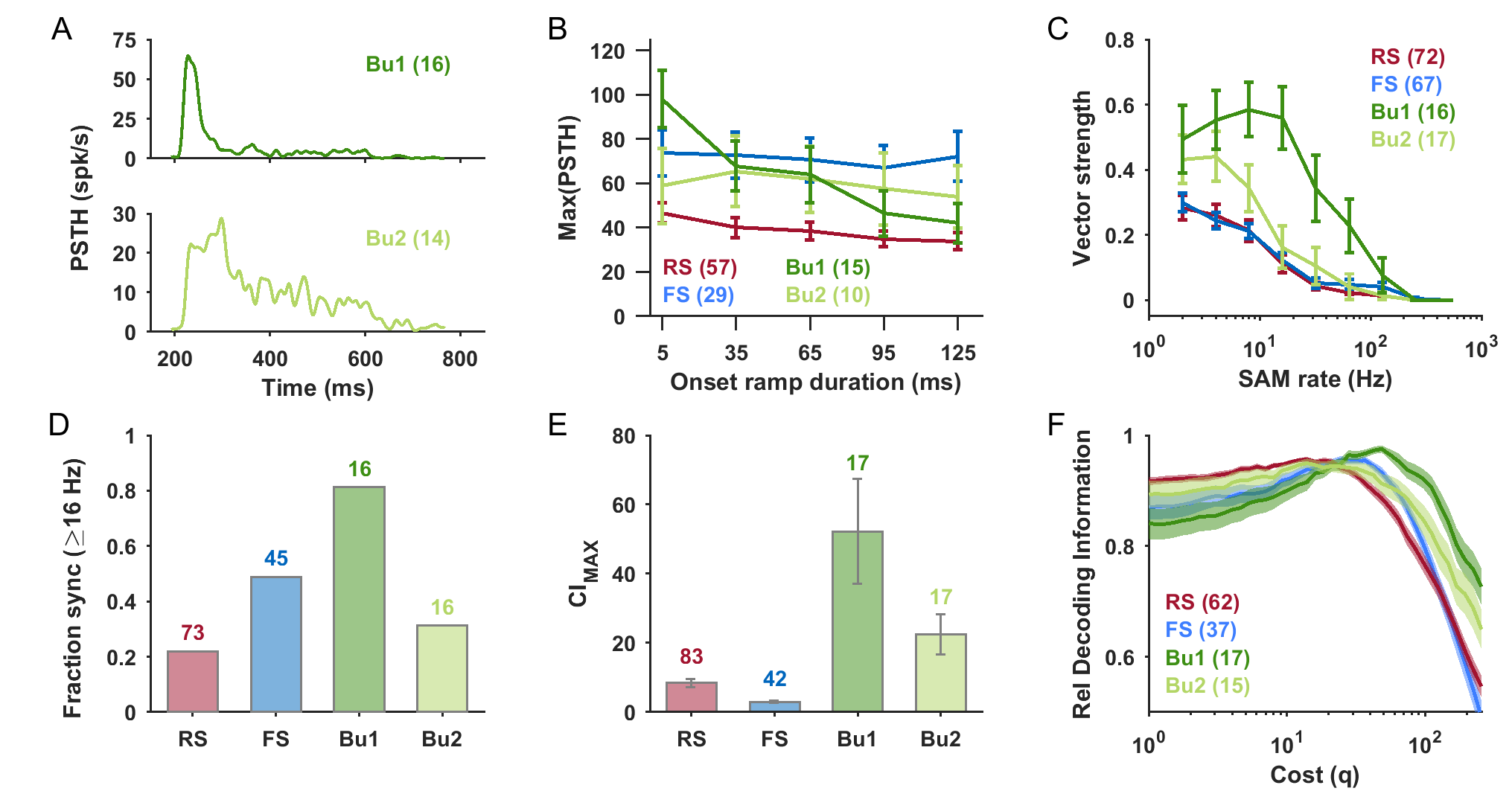

Supplement: S4 Fig — (A) Responses to 400 ms long synthetic stimuli in Bu1 units have shorter latency, higher peak firing rate, and more complete and rapid adaptation than responses in Bu2 units. (B) Bu1 unit maximum firing rate was sensitive to the rate of sound onset. (C) Bu1 unit VS was higher than Bu2 VS and peaked at intermediate SAM rates. (D) A majority of Bu1 units were synchronized at 16 Hz or higher SAM rate, in contrast with RS, FS, and Bu2 groups. (E) CI was highest for Bu1 units, indicating a tendency for spikes to occur at nearly the same time on each repetition of the vocalization stimuli. (F) This tendency is also reflected in the Bu1 group’s right shifted (toward temporal encoding) H versus q curve for decoding based on the Victor–Purpura spike distance metric. Data underlying this figure can be found in S2 Data. CI, correlation index; FS, fast-spiking; ISI, interspike interval; PSTH, peristimulus time histogram; RS, regular-spiking; SAM, sinusoidal amplitude modulation; VS, vector strength. (TIF) [file pbio.3001642.s004.tif]

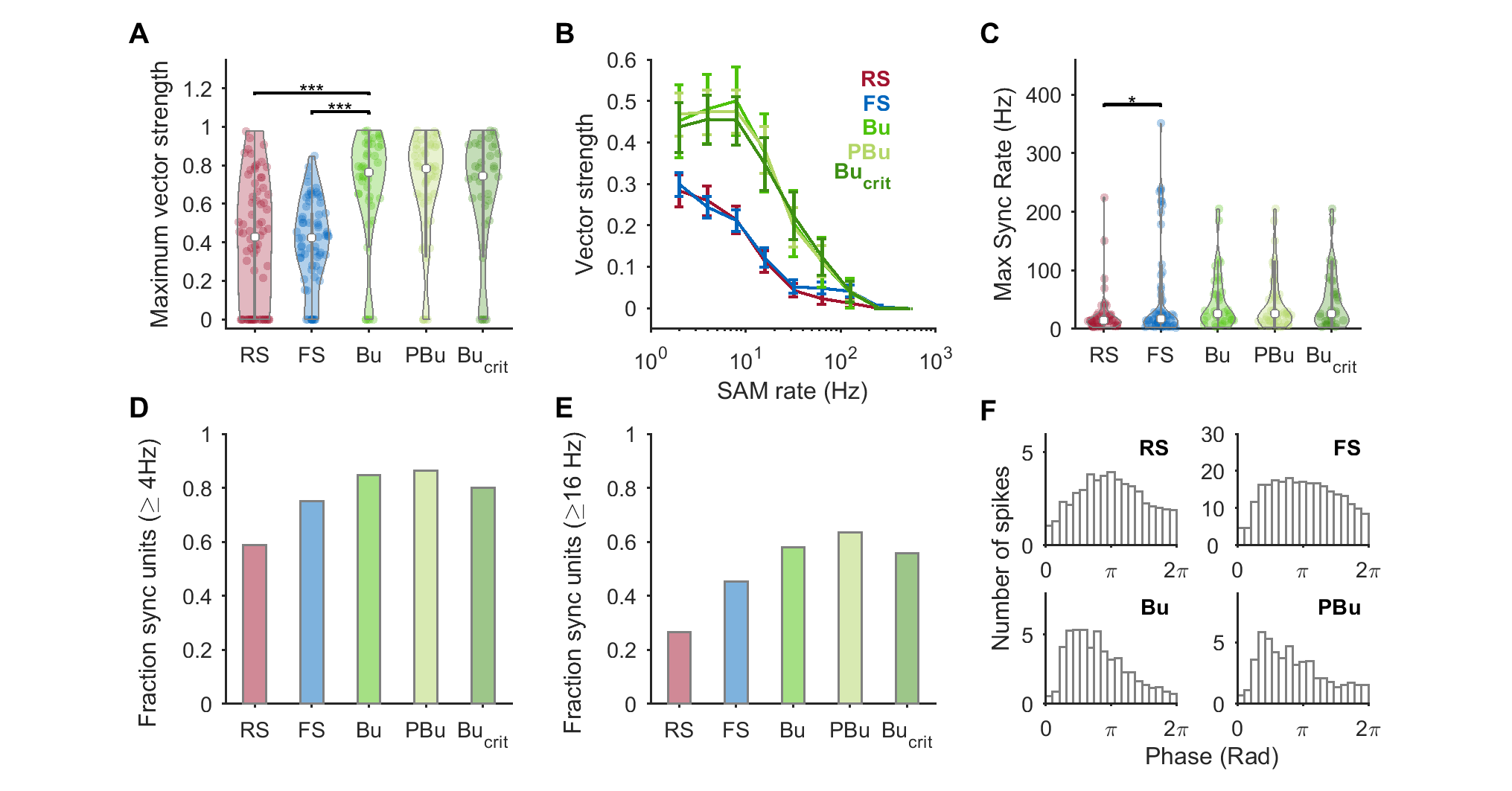

Supplement: S5 Fig — Same plots as Fig 6, but using unit type labels from the clustering analysis rather than the labels generated by criteria. Bursting units identified by 3 methods are shown for comparison: Bu (from GMM), PBu (from prestimulus logISIdrop), and Bucrit (from method of criteria, Bu1 and Bu2 combined). (A) Violin plot of maximum VS for RS (74), FS (65), Bu (40), PBu (45), and Bucrit (35) units. (B) Mean VS versus SAM modulation rate. (C) Violin plot of maximum synchronized rate for each unit type. (D) Fraction of responsive units that were synchronized at or above 4 Hz. (E) Fraction of responsive units that were synchronized at or above 16 Hz. (F) Average period histograms for stimulation at 2 Hz. Data underlying this figure can be found in S2 Data. Bu, bursting; FS, fast-spiking; GMM, Gaussian mixture model; RS, regular-spiking; SAM, sinusoidal amplitude modulation; VS, vector strength. (TIF) [file pbio.3001642.s005.tif]

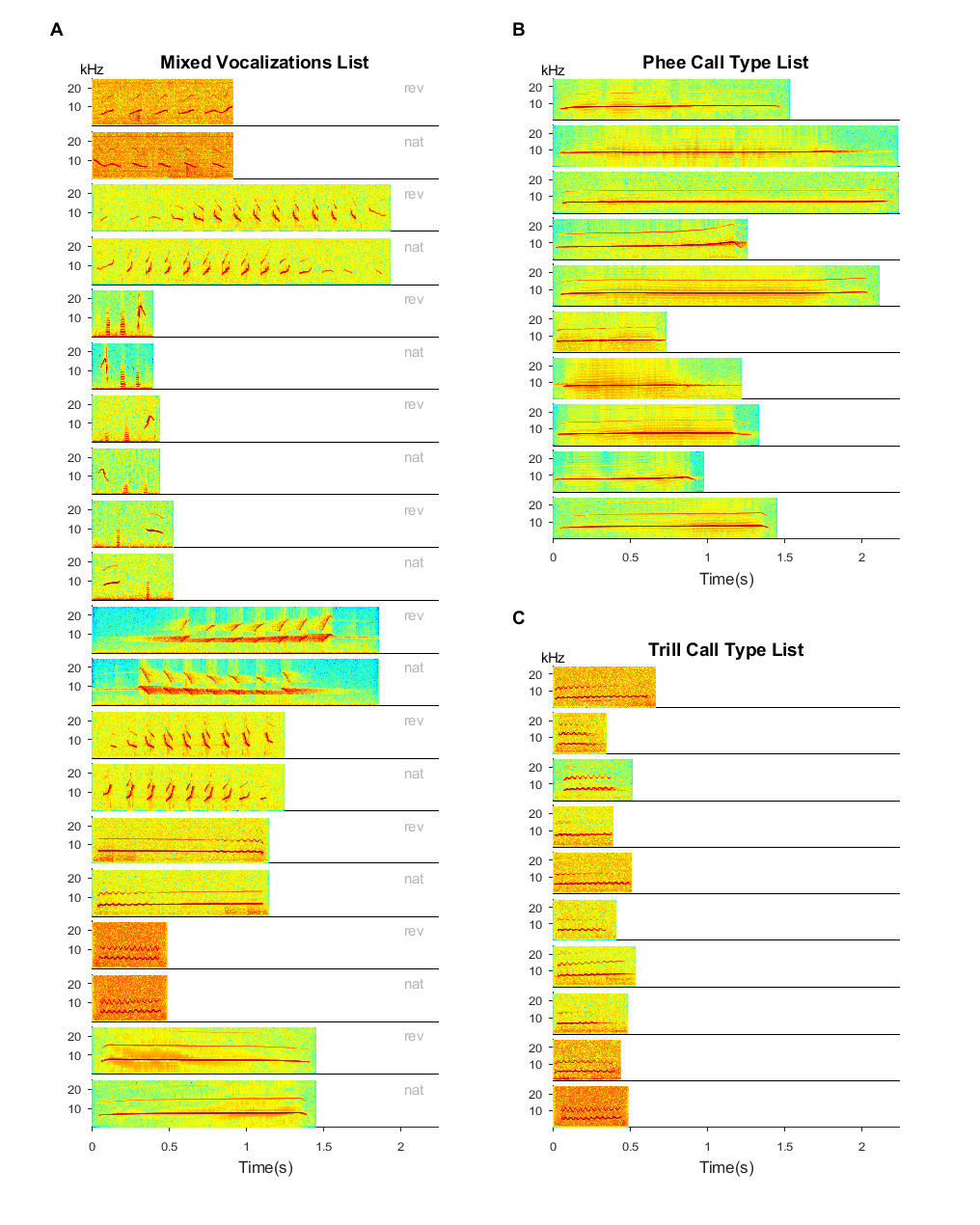

Supplement: S6 Fig — (A) The standard “Mixed Vocalizations List” included 10 call tokens in natural (“nat”) or and time-reversed (“rev”) orientation. For detailed descriptions of call types and compound calls, refer to [114]. In some cases, we also played lists of example tokens of the same vocalization type, such as the “Phee Call Type List” (B) and “Trill Call Type List” (C). (PNG) [file pbio.3001642.s006.png]

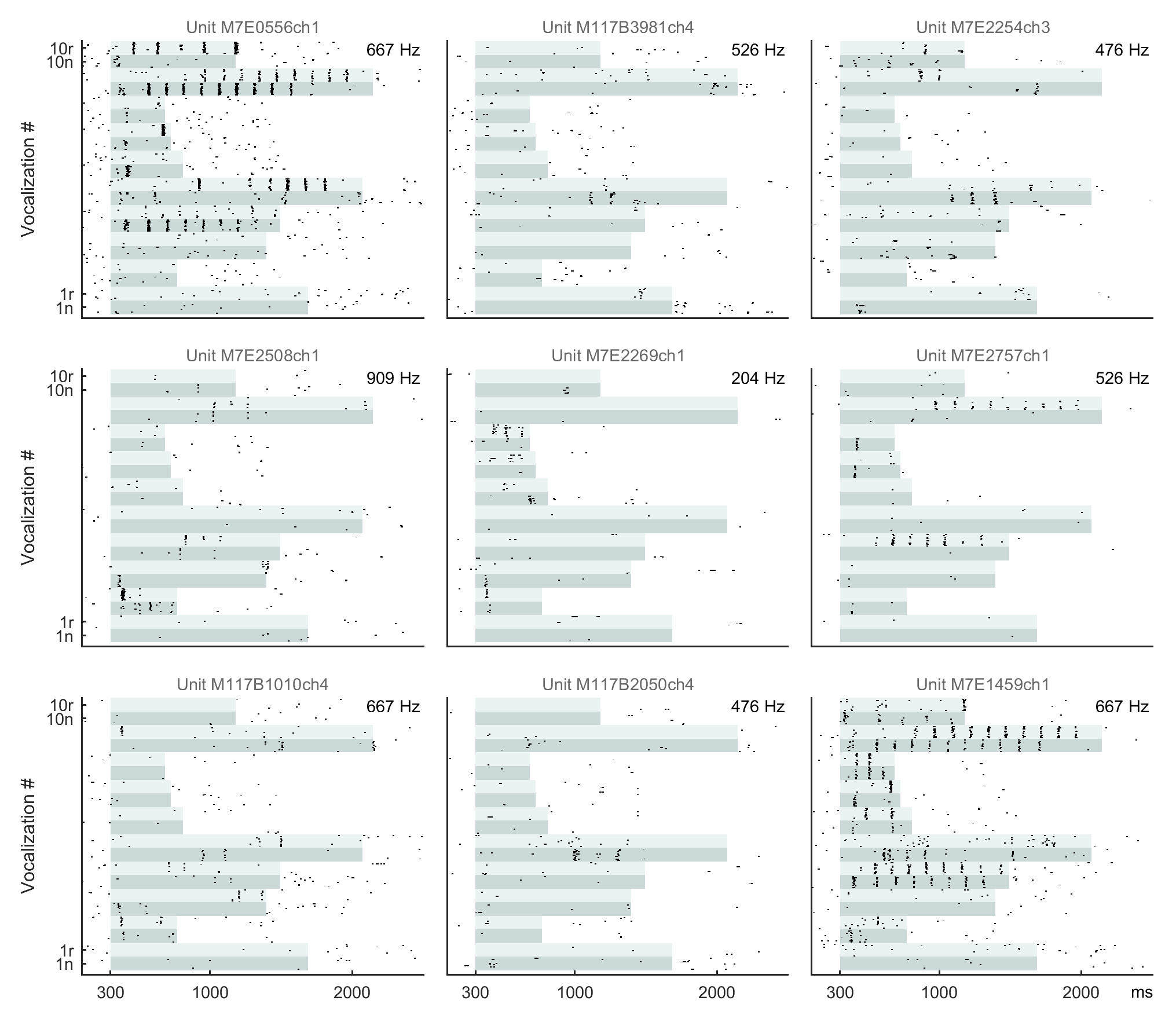

Supplement: S7 Fig — Examples of diverse precise responses to vocalizations from bursting units (intraburst frequency shown in top right corner). Alternating light aqua shading indicates the stimulus duration. Data underlying this figure can be found in S2 Data. (TIF) [file pbio.3001642.s007.tif]

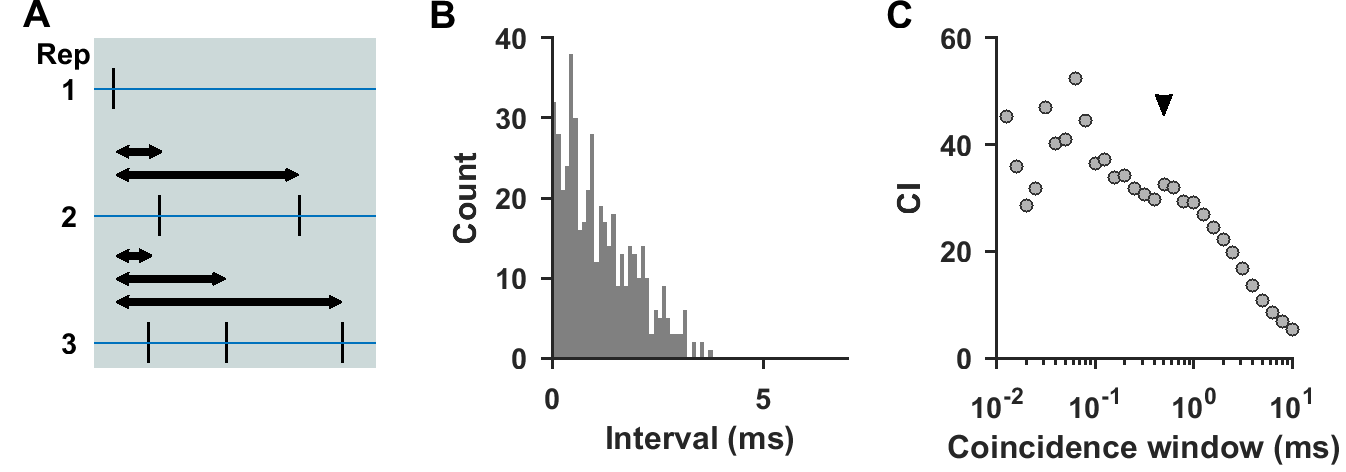

Supplement: S8 Fig — (A) The SAC was calculated as the all-order ISI histogram between spikes in 1 repetition and spikes in all other repetitions of that stimulus. “Shuffling” by excluding within-trial intervals removes the effect of the refractory period and direct effects of bursting. (B) An example of the SAC calculated from the response of a bursting unit to a marmoset trill vocalization, cropped to show short time scale autocorrelation. There was a strong tendency for spikes to occur within milliseconds of each other in the stimulus time frame across repetitions. (C) From the SAC, we can calculate the CI, a normalized measure of the prevalence of “coincidences,” or intervals smaller than a particular coincidence window (ω) [42]. For very small coincidence windows, we see a higher level of noise. For large windows, the coincidence “density” falls off. We chose to calculate the CI as the average of the 5 values around ω = 0.5 ms (black arrowhead). The CI measures the tendency for spikes to occur at the same time(s) within the stimulus, can be seen as a generalization of VS to aperiodic stimuli, and is scaled to account for firing rate, stimulus duration, number of repetitions, and ω. Data underlying this figure can be found in S2 Data. CI, correlation index; ISI, interspike interval; SAC, shuffled autocorrelogram; VS, vector strength. (TIF) [file pbio.3001642.s008.tif]
