# Supplementary material for: Distinct neuronal types contribute to hybrid temporal encoding strategies in primate auditory cortex
Source: PLoS Biol. 2022 May 25;20(5):e3001642. doi: 10.1371/journal.pbio.3001642 (PMC9132345; doi:10.1371/journal.pbio.3001642)
Supplement: Attachment — Submitted filename: Responses to reviewers.docx [file pbio.3001642.s011.docx]

Responses to Reviewers’ Comments (PBIOLOGY-D-21-03075R1)

We thank the reviewers for their constructive and helpful comments. In the revised manuscript, we have carefully addressed all concerns raised by the reviewers. The following is a point-by-point response to the comments by the reviewers. All figure numbers referred to are those of the revised manuscript. The reviewers’ comments are cited below, followed by our response in red. Changes made in the revised manuscript are marked using track changes.

**List of new or revised figures:**

Figure 2 (added marginal histogram for all units including uncategorized)

Figure 6 (added aligned rasters)

Figure 8 (new)

Supplementary Figure S2 (new)

Supplementary Figures S3-S8 (renumbered, formerly S2-S7)

Supplementary Figure S3 (added mean burst length)

Supplementary Figure S6 (added spectrograms for call type lists)

Reviewer #1: Overview
This study characterizes response properties of single neurons in the auditory cortex of awake marmosets, and concludes that there are two distinct functional classes, Regular Spiking and Bursting cells, the latter displaying superior encoding of rapid temporal features. The strength of the study is the breadth of descriptive and quantitative physiological assessments. However, significant weaknesses are that neural responses to natural stimuli (i.e., vocalizations) are poorly described, the data is pooled across many functionally distinct regions of auditory cortex, and the absence of a population analysis that evaluates how each sub-population contributes to encoding. These shortcomings can be addressed without the need for additional data collection.

Thank you for the careful reading of the manuscript and constructive comments to strengthen the paper. We have tried to address the concerns outlined below with additional analyses and text.

Main comments:

(1) The manuscript, as written, is narrowly directed to the field of auditory neurophysiology, and may not be of interest to a broader audience. Therefore, a much broader rationale for these studies should be added to the Introduction. If possible, the experiments should be motivated by a strong hypothesis. As written, the main objective is to characterize spike categories and response properties, which comes across as a somewhat incremental advance over the authors' previous publications on temporal versus rate coding in auditory cortex.

We hypothesize that neuronal types contribute in different ways to stimulus encoding, may show specializations and trade-offs in order to do so, and should be considered separately in analysis. These ideas have been added to the Abstract, Introduction, and Discussion and hopefully will improve the appeal of the article to a broader audience. We’d also argue that showing the relationship between baseline properties and a fundamental computational dichotomy, and showing another functional purpose for bursting are of interest to the general neuroscience community. While our lab has been studying temporal versus rate coding in auditory cortex for many years, this is the first study that attempted to relate neuronal types to neuron’s response firing patterns which is a significant step forward in this line of work in awake monkey auditory cortex studies.

(2) Since different regions of the auditory cortex are known to have different response properties (e.g., Bendor & Wang, 2008), it is essential to divide the data by electrode recording location (lines 431-2: A1, R, RT, CM/CL, anterior secondary regions). Of equal importance is the recording depth and putative laminar distribution. It is possible that the distribution of response classes, especially the Bu cells, differ significantly between auditory cortical regions and/or depths which could alter the interpretation.

We thank the reviewer for raising these very important questions! Please see the new **Supplementary Figure S2**. We have added new analyses in this figure to show the distributions of the units and their properties along the lateral sulcus and across recording depths. Anterior secondary areas are mentioned because it is a bit unclear where RT stops and the next area begins at the low-frequency pole of the descending best-frequency gradient. Our recordings did not venture past the low-frequency extrema. We do not observe strong associations between unit type and best frequency or recording depth (although our depths were superficially biased due to the superficial approach and long recording times). The topographic distribution of Bu1 units is interspersed among the other unit types. The relatively small number of bursting units means that we cannot definitively establish if there are distribution differences along any of these dimensions, but it seems unlikely that they present confounding explanations for the sizeable unit type differences in encoding that we saw. Critically, projecting the units onto the sulcal axis reveals that CI_max_ values were typically much higher for Bu1 units than other units at the same anterior-posterior position. The single tungsten electrode method we used in the present study has limited power in mapping across cortical surface and depth. Future experiments with multichannel / laminar probes may be better suited to explore those issue in depth (no pun intended!).

We do acknowledge in Discussion that these and other factors likely also affect the neuron’s time scale.

(3) A full and clear description is needed for the vocalization portion of the study:
(a) As presented, four different call types are shown (phee, trill, twitter, tsik) in Supplementary Figure 5, but only responses to "phee" and "trill" appear to be presented (Figure 6), or discussed in the text. Either clarify where each call type is presented, both in the text and in the Figure legends, or remove calls that were not used in this study.

We thank the reviewer for raising these issues. We have tried to improve the clarity regarding the vocalization data and analyses. There were two kinds of stimulus panels delivered: **1)** The “Mixed Vocalizations List”, as shown in Figure S6A (formerly S5), of 10 vocalization tokens in forward and reverse, including the four call types mentioned as well as compound calls and other calls. **2)** “Call Type Lists” with example tokens of only one call type (such as *phee*). The spectrograms for the *phee* and *trill* call panels have been added to Figure S6B-C.

The “Mixed Vocalizations List” was used for all quantitative analyses, such as CI_max_ calculation, spike distance metric calculations, and population decoding.

The examples from the “Call Type Lists” shown in Figure 6 qualitatively illustrate differences between unit types that are present even when comparing responses to the same vocalization type. They also demonstrate how bursting or RS responses to vocalizations follow from responses to sustained and temporally-modulated simple stimuli (shown in the preceding two figures).

The Results section has been updated to further clarify this.

(b) Which call types are shown in Figure 7 and Supplementary Figure 6? The vocalization used for every raster should be clear to the reader.

We have updated the Figure captions to be more clear and added the spectrograms for all call stimuli shown to Fig. S6.

It would be helpful to see the firing rate for each response type to the four call types (i.e., spectrogram of call with associated rasters).

Agreed, it is natural to want to line up responses with stimuli. However, it would require a lot of space to show the responses to each stimulus in the “Mixed Vocalizations List” and each of the “Call Type Lists” for each unit. Fig. 6 has been updated to show example spectrograms and corresponding rasters for *phee* and *trill* calls.

(c) Figures 6, 7, and S6 are difficult to interpret, especially given the absence of a full description in either the text or the Figure legends. Are the light aqua/grey bands indicating call duration? For Figure 6, what do each of the light aqua/grey bands represent (does each band correspond to 10 reps of a different phee call)? Are neurons recorded in the same cortical region? The same putative layer?

The captions have been amended, e.g., “The stimulus duration is indicated with alternating aqua shading, with each band corresponding to all ten repetitions of the given stimulus (stimuli were presented in interleaved order).”

The region and layer issues were addressed above – the units shown are not taken from a particular location, but are, we believe, representative. The quantitative analyses show sizeable consistent effects on temporal response properties that agree with the qualitative observations, whereas we do not see such consistent differences in region, depth, or best frequency between unit types.

(d) It is difficult to distinguish the alternating rasters for the normal and the reversed calls (Figure 7D, Figure S6).

The colors have been adjusted to increase the contrast.

(4) All of the recordings are collected passively. Given that marmosets are a highly social species, and that auditory cortical responses are known to be modulated by active engagement in an auditory behavioral task, it is not clear how to interpret the data, especially whether the distribution of cells types and responses would differ while animals were engaged in a social interaction, or were actively discriminating between stimuli. Although the data set will not change, it is important that the Discussion present an evaluation of this issue.

It would be interesting to see how engagement interacts with unit type, although the present data cannot help answer that question. A section was added to Discussion about these possibilities.

(5) To better assess the contribution of each response type, it would be valuable to implement a population decoder. This would permit the one to include or exclude RS or Bu cells, and test for call categorization. It is not clear from the current analysis how well each class, alone, would perform. Furthermore, given that FS cells are putative inhibitory interneurons, and would not be expected to contribute to downstream decoding, a population decoder would not include this response type. An analysis of this sort would help the manuscript shift from a descriptive study to a more hypothesis-driven one.

What an interesting suggestion. We implemented a simple decoder in the new **Figure 8**. While we were hoping for a synergistic effect of including both types of units, in fact, Bu units performed incredibly well per unit relative to RS units for decoding with time binned responses, while the mixture of unit types performed intermediate to the two. We tried a few simple computational experiments too and the results are shown in Fig. 8. Intriguingly, this result suggests that Bu units form one of the groups seen in an earlier population decoding study of macaque auditory cortex (Ince et al., 2013). However, we caveat the interpretation that Bu units are “privileged” as it is not surprising that a temporally precise, relatively isomorphic representation of the stimulus can be used to decode the stimulus with fewer units.

Minor comments:

(1) Write out abbreviations in Methods and Details section. For example: Line 431: A1, R, RT, CM/CL; Line 439: SAM; Line 573: AIC, BIC; Line 544: GMM

Added to Methods, also see caption for the newly added Fig S2.

(2) Methods: Would be helpful to define best frequency and bandwidth tuning for the general reader

Added description as follows: “Synthetic bandpass noise or tones at the center frequency, sound level, and bandwidth that produced the maximal driven firing rate (referred to as best frequency, level, and bandwidth) evoked robust sustained responses in the RS and FS units, while less optimal stimuli produced less sustained responses (Fig. 1A).”

(3) Figure 1: Panel "D" is mislabeled as "B"

Good catch – fixed.

(4) Figure 1: The label for Panel "E" is missing

Fixed.

(5) Figure 1: Some of the panels are missing y-axis units. E.g., For panel A, what is the y-axis? Trial number?

Since the labels for Figure 1 are already rather cluttered, I have put the description in the caption: “Stimulus duration is shown in light aqua, with each band encompassing ten trials; adjacent stimuli in (E) are shown in alternating shades.”

(6) Figure 4: What are the units for the heat map? Firing rate?

It is max-normalized smoothed firing rate. Caption has been updated.

(7) Supplementary Figure 1A: Missing x-axis units and labels

X-axis ticks and labels added.

(8) For the raster plots, it's difficult to see the gray bars, especially when showing the responses to 'reversed' vs 'normal' vocalizations (e.g., Figure 7)

The contrast has been increased.

(9) Figure 7: Write out what the "r" and "n" is in the figure legend.

This was described in Figure 1 legend and it is now also described in Figure 7 legend.

(10) Supplementary Figure 5: Which spectrograms correspond to which call types? Labels would be helpful.

The call types are tokens including the four common types previously mentioned, as well as compound calls and other call types. Call types are described in more detail in Agamaite et al., 2015. Here the call category is not critical; the tokens just represent a set of relevant natural stimuli.


Reviewer #2:

In their manuscript entitled "Neuronal types contribute to hybrid temporal encoding strategies in primate auditory cortex", Liu and Wang investigate whether different subsets of neurons - approximately classified by waveform/activity properties - in the auditory cortex have different roles in encoding sounds, in particular vocalizations. The manuscript is approaching an interesting scientific question using rigorous methodology and is executed very well, both on the level of the writing and illustrations. There, however, remained a number of important questions to be addressed (see below) which may require substantial additional analyses.

1) The Methods section omits information about the detailed recording procedures, pointing to previous published work. While this is generally fine, for the present results it would be highly relevant to know the location of recordings, in particular in which areas/subareas were the different neuronal types localized? Was there any area-related grouping of the units, or were they distributed homogeneously among the areas? Was there any relation to preferred frequency/tonotopy? Was there any preferred depth/layer where the different groups of neurons were localized? The revised version should include an analysis of the response properties/classifying metrics/classes as a function of area/layer/tonotopic location, to relate the current findings to previous work.

We thank the reviewer for raising these questions! Details about the recording locations are updated and described in Methods under the section “Electrophysiological Recordings.” To help readers more clearly understand the relationship between unit type and location, as well as other properties such as recording depth or best frequency, we have added **Supplementary Figure S2**. In short, we do not find gross systematic relationships between unit type and these properties, and we see the difference in CI_max_ values between Bu1 and other unit types all along the anterior-posterior axis. It also seems unlikely that properties such as lack of a sustained response would be due to laminar position. However, to more precisely characterize distributions, future studies could use methods such as laminar probes.

2) Relation to other response properties: Currently, the analysis/manuscript is focussed on the specific response properties of the units, however, does not relate these results to other, more basic properties, in particular classical characterizing metrics such as STRFs, which would link the current results better to previous work. Given the time-locked response properties of the Burst neurons, an average, CF centered STRF of the groups would likely show that many clear STRFs are originating from the bursting groups. This would help explain the varying levels of SNR typically found in STRFs from auditory cortex.

The relationship between STRFs and intrinsic neuronal type is an interesting question. However, our synthetic stimuli consisted of simple tones and noises and were not designed to sample across spectro-temporal modulation space and were therefore not suited to estimate STRFs. Our vocalization stimuli had very particular correlational structure and frequency content and would be expected to only sparsely sample the space as well. Furthermore, it is not obvious what one might expect to see. On the one hand, Bu1 units were often relatively easy to drive and their responses are well time-locked, which should support robust STRFs. On the other hand, their slope-dependent behavior appears nonlinear, so STRF predictions may be inaccurate as a result. Consider the response of Bu1 units to SAM stimuli – a smooth oscillation is translated to a single time-locked burst. It is not clear that firing rate is even an appropriate description of this response.

The issue is certainly worth future exploration, but at this point we prefer to just speculate as it is not critical to the conclusions of this paper.

3) Scientists tend to classify their results in order to simplify the results. While this is a useful practice, it sometimes can mistake a continuum for a set of classes. While the presented data leave no doubt about a wide range of expression of certain response properties (i.e. bursting phenotype/phase-locking in this case), it remained in my opinion inconclusive whether there are really identifiable classes.

That’s a reasonable point and it is certainly possible there is a continuum in the cell type data we tried to classify. We now raise the issue in Discussion. This is a perpetual question with neuronal type studies, even when molecular profiles are considered (e.g., Berg et al., 2021; Gouwens et al., 2019). We know that regular-spiking and fast-spiking classes correspond to excitatory and inhibitory neurons, which have distinct embryonic origin. In terms of RS versus bursting neurons, other recent studies have similarly found multiple electrophysiologically-separable populations (including bursting types) with corresponding differences in other functional properties (e.g., Trainito et al., 2019; Onorato et al., 2020). Transcriptomic and morphological analysis combined with electrophysiology also support distinct bursting and regular-spiking populations (González-Burgos et al., 2019 and Berg et al., 2021). It is biologically plausible that these represent distinct groups. For instance, the Col22A1 group seems relatively well segregated in UMAP embedding (Berg et al., 2021).

Ideally, with two populations that are easily distinguished along a single axis, the dip test could be used. However, we don’t think it’s suitable for separating bursting and RS units, nor should it be the gold standard for multi-dimensional separation (see below).

Our Gaussian Mixture Model selected three main components as the best explanation for the observed density of response properties. If bursting and RS units formed an elongated distribution of a single population, one might expect it to instead fit two components rather than three. Therefore, our data suggest that indeed there are discrete groups. However, it would be interesting to see as more molecular and morphological data come in whether that holds up.

As the authors note themselves, " Therefore, if bursting and non-bursting units were combined, the resulting histogram may not appear bimodal", and if I interpret this sentence and the graphs correctly, the neurons that remained unclassified would in addition fill the putative dip between the putative classes (see also Fig. 2B/3B). The logic for using the dip test was inverted here (applied after selecting classes), which - given the current criteria - would be biased to find dips in many single-class or continuous distributions. By eye, if the marginal histogram in
Fig. 2B included the non-classified units, I would estimate it would not show a (significant) dip. I think the authors should therefore (1) either not make the claim that these units are 3 separate classes and instead argue for different response properties of units along a continuum, (2) provide stronger support for separable classes of neurons, e.g. either by genetic identification/biochemical labelling or identifying a combination of dimensions where the projected density of response properties passes the dip test (this might also be the case for the Fig. 3B, but I could not find a corresponding claim/analysis in the text).

The quoted statement was used to point out that seeking bimodality of a *single* feature (e.g., spike width) is often not a good strategy in a situation with multiple overlapping populations. For instance, in Trainito et al., 2019, the authors state: “In our data, only the repolarization time showed clearly two distinct modes (see marginal histograms in Figure 1B), whereas trough-to-peak duration likely consisted of even more latent components. Together, these measures allowed for defining four bivariate clusters that were less discriminable when projected only onto one dimension.” The Results text has been amended to clarify this point.

We used criteria on *spike timing* to first separate out bursting units. This helped reveal bimodality of *spike widths* in the remaining mixture because bursting units had intermediate spike widths (Fig. 2D). Since spike width was not used to separate out bursting units, this is not circular. No dip test was performed in Fig. 2B (showing spike timing properties). The evaluation of the dip test was on the properties in Fig. 2D (showing spike waveform width properties). Furthermore, if the question is whether RS and bursting units are discrete, FS units would have to first be semi-arbitrarily removed from the peakISI distribution. Later, the dip test was also applied to the log(ISI) distributions of individual units, which is distinct from the distribution of peakISI values across units.

We now show the marginal histogram for all units in Fig. 2B, including unclassified units, as gray shading. The remaining non-bursting units (all within the purple cloud of Fig. 2B) showed bimodality in spike waveform properties in Fig. 2D, per the dip test.

The Gaussian Mixture Model in essence provides an objective multidimensional separation of the groups. A reasonable separation can be seen already with the first two principal components, but in two orthogonal directions, so I’m not sure how one could perform a dip test (it does not look unimodal in 2D as it is L-shaped).

In association with the question above, the authors could pursue the question of whether classes or a continuum would be better suited for en-/decoding of naturally occurring sounds/vocalizations (likely continuum). While this appears to be outside the scope of the present article, it would provide a strong motivation for the existence of the observed range of response/coding properties.

This is an interesting question. To rigorously address the contribution to en-/decoding, one would make some assumptions to create model neurons with particular receptive fields and specify what parameters are varied in a continuum, which may be done in future modeling studies.

4) Availability of the Data/Analysis Code: In their submission information, the authors indicate that the data is fully available, however, no additional information is provided. In particular during the review process the availability of the (near raw) data and the analysis code would be useful. It could e.g. be made available to reviewers confidentially during the review process, and later openly to all readers.

Analysis code has been made available on Gitlab. Due to there being no field in the submission form for this information, we were advised to put the details in the Methods for the moment.

Data has been made available in the Supplemental Information per journal guidelines.

5) Bursting or 'Doublet Neurons': It was hard for me to assess what the number of spikes in a burst typically was, from the information in the manuscript. Are these dominantly doublets or triplets or even longer sequences? To clarify this, the revision should include a histogram of the number of spikes in a burst, and then relate this finding to bursting cells in other systems (extending/modifying the excellent discussion on this topic).

An example of bursts can be seen in the insets in Fig. S1, and Fig. S3 has been modified to show violin plots of “Mean burst length” to provide the distribution of this value per unit. Although the interpretation of this value is less clear for RS and FS units, it is constructed to capture burst length for Bu units. Two to three spikes is typical. This has been added to Results and a comparison to other studies of cortical bursting neurons is included.

Minor:
- Please include the Bu1/Bu2 grouping into Fig. 2.

We thought this would be confusing because the Bu1/2 distinction is not introduced until the section on responses to varying duration stimuli where we present some rationale for the subgroups. The distinction has now been added to the scatterplots as green triangles versus diamonds, as was done for Fig. 3B.

- Please include the marginal across all units in Fig. 2B (right subpanel)

This has now been added as gray shading to Fig. 2B. But again, the dip tests were not performed on this distribution.

- An interesting general reference for the introduction: <https://pubmed.ncbi.nlm.nih.gov/30034330/>

Thanks! We have added it to the Discussion.

Reviewer #3: No comments (accept).
